# MNAzyme-Assisted Nucleic Acid Lateral Flow Assay for Cost-Effective, On-Site Mercury Detection

**DOI:** 10.3390/bios14100454

**Published:** 2024-09-25

**Authors:** Seok Hyeon Kim, Yujun Kim, Seokjoon Kim, Eun Sung Lee, Byung Seok Cha, Ki Soo Park

**Affiliations:** Department of Biological Engineering, College of Engineering, Konkuk University, Seoul 05029, Republic of Korea; sh7041@konkuk.ac.kr (S.H.K.); yujun005@konkuk.ac.kr (Y.K.); ghjghy@konkuk.ac.kr (S.K.); afish94@konkuk.ac.kr (E.S.L.); cbs934@konkuk.ac.kr (B.S.C.)

**Keywords:** mercury, detection assay, MNAzyme, NALFA

## Abstract

Mercury ions (Hg^2+^) are toxic heavy metals present in the environment that pose significant health risks. An advanced detection system could allow for a prompt response and alleviate serious damage to humans. In this study, we developed a cost-effective, on-site detection method for Hg^2+^ using a multicomponent nucleic acid enzyme (MNAzyme)-assisted nucleic acid lateral flow assay (NALFA). The MNAzyme, which was engineered to contain thymine–thymine mismatches, is responsive only to the presence of Hg^2+^ and exerts efficient cleavage activity on substrates that can be captured by the NALFA strip, and thus the proposed system enables the visual detection of Hg^2+^ in the NALFA strip. Our assay demonstrated sufficient detection sensitivity and specificity to meet the WHO standards, offering a good practical alternative for rapid environmental and public health monitoring.

## 1. Introduction

Mercury is a heavy metal that is naturally present in air, water, and soil, and is released into the environment through volcanic activity, rock weathering, or human activities [1]. Mercury ions (Hg^2+^), one of the most common and stable forms of mercury, are a persistent toxic pollutant that can accumulate in living organisms through the food chain [2]. Long-term exposure to an environment where mercury is present can lead to serious health problems, including brain damage, kidney failure, and chronic DNA damage [3,4]. Thus, there is a high demand for detection systems capable of detecting Hg^2+^.

Current Hg^2+^ analytical methods include inductively coupled plasma mass spectrometry (ICP-MS), inductively coupled plasma atomic emission spectrometry (ICP-AES), inductively coupled plasma optical emission spectrometry (ICP-OES), cold vapor atomic fluorescence spectrometry (CV-AFS), and high-performance liquid chromatography (HPLC). Although these methods are accurate and have sufficient sensitivity to meet the World Health Organization (WHO) standard of 6 ppb, they require bulky and expensive equipment, specialized spaces, and trained operators, rendering their implementation in the field challenging [5,6,7,8,9]. To overcome these shortcomings, various approaches have been developed based on the fact that Hg^2+^ can specifically bind to thymine–thymine (T-T) mismatches to form T-Hg^2+^-T pairs, which are more stable than Watson–Crick adenine (A)–thymine (T) pairs and do not affect the thermal stability of double-stranded DNA (dsDNA) [10,11,12].

RNA-cleaving DNAzymes, which are composed of a conserved catalytic core and two substrate-binding arms, can catalyze RNA cleavage reactions on substrates with multiple turnovers in the presence of metal ion cofactors (e.g., Mg^2+^ or Mn^2+^) [13]. They have gained attention in bioanalysis because of their advantages, such as high stability, strong catalytic activity, ease of synthesis, functionalization, and modification [14]. Furthermore, the multicomponent nucleic acid enzyme (MNAzyme), in which the catalytic core of DNAzyme is split into two subunits and a functional catalytic core capable of cleaving multiple substrates is formed only in the presence of an assembly facilitator, such as a target nucleic acid molecule, has been reported for more widespread applications, including biosensors [15,16]. A representative example is found in a system for the detection of Hg^2+^, in which the target induces the formation of a functionally active DNAzyme or MNAzyme by forming stable T-Hg^2+^-T pairs [17,18,19,20]. However, it relies on fluorescence signaling and requires the expensive dual modification of the fluorophores and quenchers, which limits its practical application in facility-limited or point-of-care settings.

In this study, we developed a nucleic acid lateral flow assay (NALFA)-based colorimetric Hg^2+^ detection system by designing an Hg^2+^-specific MNAzyme, in which T-T mismatches were rationally placed next to the split catalytic core sequence of the MNAzyme. The presence of Hg^2+^ induces the formation of an active MNAzyme that catalyzes multiple substrate cleavage reactions. Importantly, the cleaved substrate was too short to generate a colorimetric signal via DNA hybridization in the test line of the NALFA strip, which could be clearly discerned by the vivid red signal in the absence of Hg^2+^. To the best of our knowledge, this is the first study to construct a NALFA system in combination with an MNAzyme for Hg^2+^ detection. Notably, the system excluded any modifications to DNA oligonucleotides, significantly reducing production and assay costs compared to conventional LFA that requires one or more haptens and interacting counterparts (e.g., biotin and streptavidin). Furthermore, the analytical results can be confirmed even with the naked eye, enabling the proposed system to serve as a simple and cost-effective monitoring tool for Hg^2+^ in the field.

## 2. Materials and Methods

### 2.1. Reagents and Materials

All oligonucleotides (Table S1) were synthesized by Bionics (Seoul, Republic of Korea), except for the substrate oligonucleotides, which were obtained from Integrated DNA Technologies (Coralville, IA, USA). The melting temperatures of the oligonucleotides were calculated using the OligoAnalyzer tool provided by Integrated DNA Technologies (https://idtdna.com/pages/tools/oligoanalyzer, accessed on 22 April 2024). Lead(II) acetate trihydrate, mercury(II) standard solution, and gold(III) chloride trihydrate were purchased from Sigma-Aldrich (St. Louis, MO, USA). Sodium chloride, calcium(II) chloride, cobalt(II) chloride hexahydrate, cesium chloride, sodium acetate trihydrate, and acetic acid were obtained from Samchun Chemicals (Seoul, South Korea). Magnesium(II) chloride hexahydrate, nickel(II) chloride hexahydrate, potassium chloride, zinc(II) chloride, barium(II) chloride, chromium(III) chloride hexahydrate, tris(hydroxymethyl)aminomethane, and Tween 80 were purchased from Daejung Chemicals and Metals (Gyeonggi-do, Korea). Manganese(II) chloride, lithium chloride, sodium selenite, and trisodium citrate dihydrate were obtained from Shinyo Pure Chemicals Co. (Osaka, Japan), Tokyo Chemical Industry (Tokyo, Japan), KANTO CHEMICAL Co., Inc. (Tokyo, Japan), and Junsei Chemicals (Tokyo, Japan), respectively. Nitrocellulose membranes (FF80HP PLUS) were obtained from Whatman (Maidstone, UK), cellulose membranes from Merck Millipore (MA, USA), backing cards from TWOHANDS (Gyeonggi-do, Republic of Korea), 1 M Tris-HCl (pH 8.0) from HanLAB (Gyeonggi-do, Republic of Korea), and ultrapure water from Koup (Suwon, Republic of Korea).

### 2.2. Urea Polyacrylamide Gel Electrophoresis (Urea-PAGE) Analysis

Reaction solution containing 10 mM Tris-acetate buffer (pH 8.0), 100 mM NaCl, 100 mM M1 DNA and M2 DNA, 10 mM MgCl_2_, and 0.5 mM MnCl_2_ was prepared, which was then heated at 95 °C for 5 min and snap-cooled on ice for 5 min. Subsequently, 100 ppb Hg^2+^ and 200 nM substrate were added. After the incubation at 30 °C for 30 min, the sample was mixed with Novex™ TBE-Urea Sample Buffer (2X) (Invitrogen, Carlsbad, CA, USA) and incubated at 70 °C for 5 min to break hydrogen bonds. Next, 10 µL of pretreated samples were electrophoresed on 14% Urea PAGE at 150 V for 50 min in 1× TBE buffer and were stained in GreenStar Nucleic Acid Staining Solution (Bioneer, Daejeon, Republic of Korea) for 10 min. Finally, gel images were acquired using the ChemiDoc system (Bio-Rad Laboratories, Hercules, CA, USA).

### 2.3. Synthesis of Gold Nanoparticles (AuNPs)

AuNPs were synthesized according to the citrate reduction method [21]. Briefly, 100 µL of HAuCl_4_ (500 mM) and 388 µL of trisodium citrate solution (500 mM) were quickly added to 50 mL of boiling ultrapure water. After the solution was incubated for 10 min, it was allowed to cool at room temperature and then stored at 4 °C for further use. The synthesized AuNPs were characterized using a microplate reader (SpectraMax iD5 Multi-Mode Microplate Reader; Molecular Devices, San Jose, CA, USA), and were found to have an absorbance peak at 520 nm with a diameter of 20 nm.

### 2.4. Modification of AuNPs with DNA

The DNA modification of the AuNPs was performed using a microwave-assisted heating and drying method [22]. In brief, 190 µL of the synthesized AuNP solution and 10 µL of Tag DNA (100 µM; Table S1) were added and mixed thoroughly by pipetting. The mixture was then microwaved for 5 to 10 min until the liquid was sufficiently evaporated. Once all the liquid was evaporated, 200 µL of ultrapure water was added and the sample was resuspended. After centrifugation at 12,400× *g* for 20 min, the supernatant was removed and the pellet was resuspended in 200 µL of ultrapure water, which was stored at 4 °C for further use. As shown in Figure S1, the absorption maxima from Tag DNA-modified AuNPs, which refer to the specific wavelengths at which a substance absorbs the most light, were red-shifted by approximately 6 nm compared to non-modified AuNPs, indicating that the Tag DNA was attached to the surface of the AuNPs [22,23].

### 2.5. Preparation of the NALFA Strip and Immobilization of Capture DNA

First, a nitrocellulose (NC) membrane (300 × 25 mm) was pasted on a backing card, where absorbent pads (cellulose membrane; 300 × 23 mm) were affixed with a 3 mm overlap with the NC membrane to facilitate capillary flow (Figure S2). After the prepared NALFA strips were cut into 3 mm wide pieces, the capture DNA was immobilized according to our own SAIoN protocol [24]. In brief, 0.3 µL of capture DNA solution (80 µM capture DNA in the test line; Table S1 and 0.8 M NaCl) was applied to the test line of the LFA strip, and the control capture DNA solution (80 µM capture DNA in the control line; Table S1 and 0.8 M NaCl) was applied to the control line of the LFA strip, respectively, and allow to dry for 10 min. Once the LFA strip was sufficiently dried, it was irradiated at 254 nm (total energy: 90 mJ/cm^2^) for 5 min using a UV crosslinker (Korea Ace Science, Seoul, Republic of Korea).

### 2.6. Hg^2+^ Detection Assay

The reaction solution containing 4 µL of 75 mM Tris-Acetate buffer (pH 8.0), 2 µL of 1 M NaCl, 1 µL of 1 µM M1 DNA and 1 µL of 1 µM M2 DNA, 1 µL of 200 mM MgCl_2_, 2 µL of 5 mM MnCl_2_, and 6 µL of ultrapure water was prepared, which was then heated at 95 °C for 5 min and snap-cooled on ice for 5 min. Subsequently, 1 µL of various concentrations of Hg^2+^ solution and 2 µL of 2 µM substrate were added and incubated at 30 °C for 90 min. After the reaction was stopped by adding 80 µL of 62.5 mM EDTA (pH 8.0), the reaction solution was diluted to achieve a final substrate concentration of 15 nM, and 3 µL of the stopped reaction solution was mixed with 15 µL of 2× running buffer (0.32 M Tris-HCl (pH 8.0), 1.2 M sodium acetate, 1.2% Tween 80), 3 µL of Tag DNA-modified AuNPs, and 9 µL of ultrapure water. Next, the NALFA strip was soaked in the reaction solution for 10 min, after which it was washed with 1× running buffer. Finally, the NALFA strip was photographed using a smartphone camera (Galaxy s20, Samsung, Seoul, Republic of Korea) at a constant distance and brightness. The color intensity was analyzed using ImageJ 1.54d software, and the background signal was subtracted.

### 2.7. Detection of Hg^2+^ in Tap Water

Tap water samples were collected from the Konkuk University campus and various concentrations of Hg^2+^ were spiked to the tap water samples. After removing the impurities using a 0.22-µm filter (Satorius, Göttingen, Germany), the tap water samples were analyzed using the Hg^2+^ detection assay described previously (Section 2.6) as well as the standard method, ICP-OES.

## 3. Results and Discussion

### 3.1. Hg^2+^ Detection Strategy

**Scheme 1:**
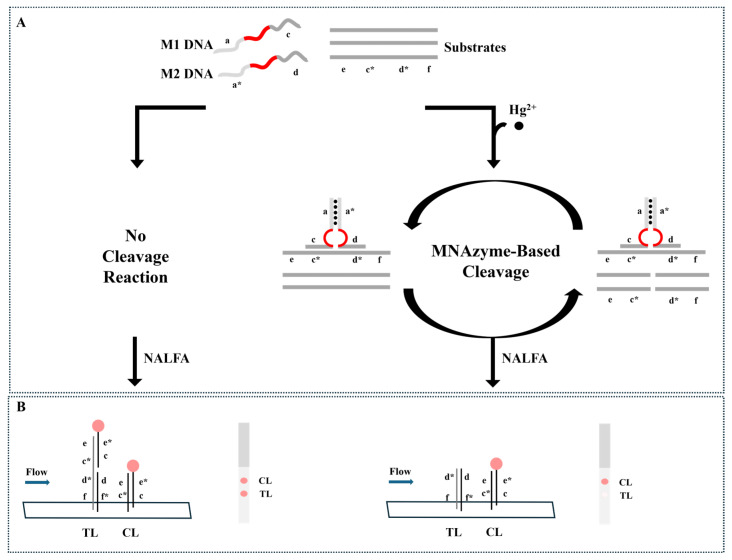
Schematic illustration of the Hg^2+^ detection assay where (**A**) MNAzyme-based cleavage reaction occurs only in the presence of Hg^2+^ and (**B**) the reaction products are analyzed by the NALFA strip. The lowercase letters indicate the specific region in the DNA oligonucleotide and * indicates their complementary sequences. AuNPs (red circles) are modified with Tag DNA using a heating and dry method, and capture DNA is immobilized on the NALFA strip using our own SAIoNs method (see the experimental details). The 5’-end (e, c*) and 3′-end regions (d*, f) of the substrate hybridize with Tag DNA (e*, c) on the AuNPs and with the capture DNA (d, f*) in the test line (TL) of the NALFA strip, respectively. Thus, the substrate, produced only in the absence of Hg^2+^, can bind to both capture DNA and Tag DNA, generating an intense color signal in the test line of the NALFA strip, which does not occur in the presence of Hg^2+^ that induces MNAzyme-catalyzed cleavage reactions. In the case of the control line (CL), the capture DNA (e, c*) hybridizes with Tag DNA (e*, c) on the AuNPs, thereby generating the colorimetric signal regardless of the presence or absence of Hg^2+^. Not all DNA oligonucleotides entail chemical modifications.

Scheme 1A shows the Hg^2+^ detection strategy, in which the RNA cleaving DNAzyme was engineered to construct MNAzymes responsive to Hg^2+^. Specifically, the catalytic core of the DNAzyme was split into two subunits, and each subunit (M1 and M2 DNA; Table S1) was designed to contain three parts: an Hg^2+^ recognition sequence containing T-T mismatches (a, a*), a catalytic core (red color), and a substrate-binding arm (c, d). In addition, the substrate (e, c*, d*, f) containing RNA in the middle where the cleavage occurs has the sequences (c* and d*) complementary to the substrate binding arms (c and d) of the M1 and M2 DNA. Since the melting temperature of the substrate binding arms (c/c* and d/d*) was calculated to be less than 25.6 °C, each subunit (M1 and M2 DNA) of MNAzyme was separately present in the absence of Hg^2+^ and was not functionally active at our reaction temperature of 30 °C. In contrast, the two subunits were assembled to form a complete MNAzyme structure in the presence of Hg^2+^, which induced the formation of T-Hg^2+^-T pairs, exerting effective cleavage activity on the substrates. Consequently, non-cleaved and cleaved substrates were produced in the absence and presence of Hg^2+^, respectively, via MNAzyme-catalyzed reactions, which could be visualized with the naked eye in the NALFA strip. As shown in Scheme 1B, the reaction products flowed through the NALFA strip, and a vivid red color signal appeared on the test line only in the absence of Hg^2+^; its signal significantly decreased in the presence of Hg^2+^. The control line was designed to generate an intense colorimetric signal regardless of the presence or absence of Hg^2+^, which was used to verify the appropriate performance of the NALFA strip. The proposed system is suitable for the detection of Hg^2+^ in the field, where the facilities are limited.

### 3.2. Detection Feasibility

**Figure 1:**
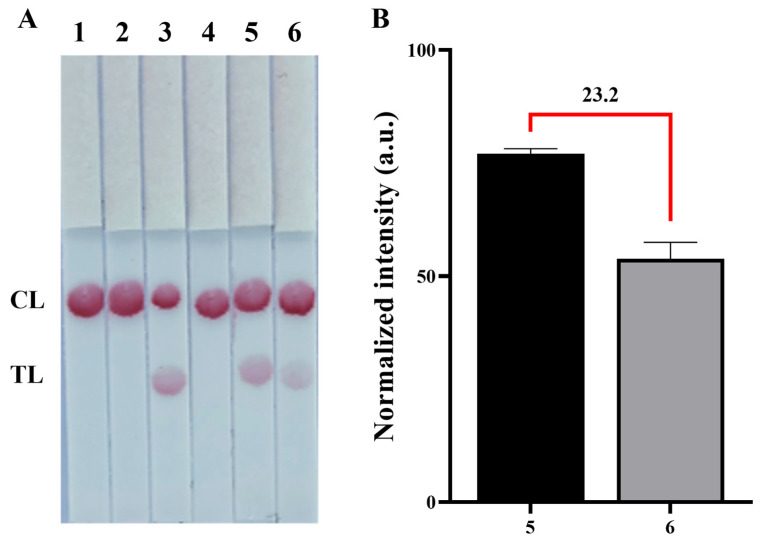
Feasibility of the Hg^2+^ detection assay. (**A**) Images of the NALFA strips. NALFA strips 1–4 are M1 DNA, M2 DNA, substrate, and cleaved substrates, respectively, and NALFA strips 5 and 6 are the reaction products where all detection components (M1 DNA, M2 DNA, and substrate) are present without and with Hg^2+^ (100 ppb), respectively. (**B**) Normalized intensity of NALFA strips 5 and 6. The number above the bar graph is calculated by subtracting the normalized intensity of NALFA strip 6 from the one of NALFA strip 5. The error bars represent the standard deviation from three independent experiments.

To validate the detection feasibility, we first investigated the MNAzyme-catalyzed cleavage reaction under different conditions using Urea-PAGE. Lanes 1 to 4 are the control samples, which indicate the location of M1 DNA, M2 DNA, substrate, and cleaved substrates, respectively, whereas lanes 5 and 6 are our reaction solutions that contain all detection components, including M1 DNA, M2 DNA, and substrate; however, Hg^2+^ is present only in lane 6. As shown in Figure S3, a specific band corresponding to the cleaved substrate appeared only in lane 6, confirming that the designed MNAzyme system was functionally active and effectively cleaved the substrate in the presence of Hg^2+^. Next, we tested whether the results of the MNAzyme-catalyzed cleavage reaction could be analyzed visually using the proposed NALFA system. As shown in Figure 1A, NALFA strips 1–4 indicate the control samples where M1 DNA, M2 DNA, substrate, and cleaved substrate were applied, respectively. (1) M1 DNA, (2) M2 DNA, and (4) cleaved substrate did not show any colorimetric signal in the test line, but (3) only substrate showed a colorimetric signal in the test line. Importantly, the results for NALFA strips 5 and 6, which contained all the detection components in the absence and presence of Hg^2+^, respectively, showed that the colorimetric signal was significantly reduced only in the presence of Hg^2+^. We also quantified the colorimetric results using ImageJ software by normalizing each value against that obtained for strip 3, where a substrate was applied. As shown in Figure 1B, the decrease in the normalized intensity was 23.2 by the presence of Hg^2+^, demonstrating the colorimetric detection feasibility of the NALFA system.

### 3.3. Optimization of the Hg^2+^ Detection Assay

**Figure 2:**
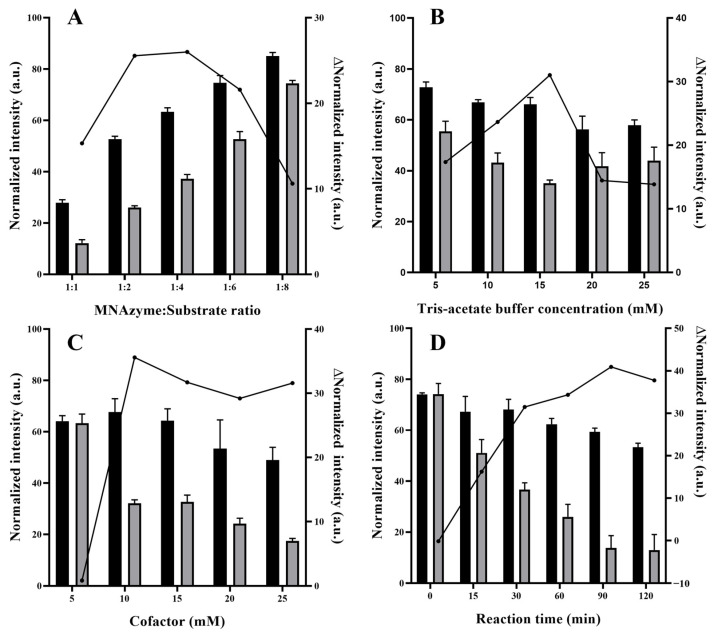
Optimization of the Hg^2+^ detection assay. (**A**–**D**) Normalized intensity at different ratios of MNAzyme to substrate, different Tris-acetate buffer concentrations, different cofactor concentrations, and different reaction times. The black and gray bars represent the absence and presence of Hg^2+^ (100 ppb), respectively. ∆Normalized intensity is calculated by subtracting the normalized intensity in the presence of Hg^2+^ (100 ppb) from the normalized intensity in the absence of Hg^2+^. In (**A**), the concentration of MNAzyme was fixed at 50 nM, while the concentration of substrate was varied from 50 to 400 nM. Error bars represent the standard deviation from three independent experiments.

After confirming that the MNAzyme-assisted NALFA system could be used for the colorimetric detection of Hg^2+^, we optimized the reaction conditions to achieve the best performance. First, we optimized the amount of substrate applied to the NALFA strip, because too few or too many substrates could generate false-positive or false-negative results. As shown in Figure S4, the colorimetric signal became stronger as the substrate concentration increased. However, the slope of the colorimetric signal increase was the largest at a substrate concentration of 1.5 nM, indicating that the change in colorimetric signal by the presence of Hg^2+^ would be the largest. Therefore, the final substrate concentration applied to the NALFA strip was selected to be 1.5 nM. Next, we optimized the ratio of Hg^2+^-specific MNAzymes to their substrates. As shown in Figure 2A, the normalized intensity was the lowest when the ratio of MNAzyme to substrates was 1:1, which might be because the substrate-binding arms of M1 and M2 DNA interfere with the binding of substrates to the capture and Tag DNA in the NALFA strip. An MNAzyme-to-substrate ratio of 1:4 showed the largest ∆normalized intensity, which was chosen for further experiments. Given that Tris-acetate buffer concentration affects ionic strength and the pH buffering capacity, an experiment was conducted to identify the optimal Tris-acetate buffer concentration. As illustrated in Figure 2B, the impact of the Tris-acetate buffer concentration was minimal in the absence of Hg^2+^, whereas the greatest reduction in the colorimetric signal due to the presence of Hg^2+^ was observed at a Tris-acetate buffer concentration of 15 mM. Therefore, a Tris-acetate buffer concentration of 15 mM was used in subsequent experiments. Furthermore, the concentration of cofactor ions (Mg^2+^) for the catalytic activity of the MNAzyme was optimized. As shown in Figure 2C, the MNAzyme did not work effectively at a Mg^2+^ concentration of 5 mM, and the ∆normalized intensity was negligible. However, the normalized intensity in the absence of Hg^2+^ decreased when it was over 20 mM, which was attributed to the undesirable hybridization between M1 and M2 DNA leading to unwanted substrate cleavage. Thus, 10 mM of Mg^2+^, which showed the highest ∆normalized intensity, was selected for further experiments. In case of Mn^2+^, the other cofactor ions for the catalytic activity of the MNAzyme, 0.5 mM was selected based on the previous report [16]. Finally, the reaction time for the MNAzyme-catalyzed cleavage was optimized. As shown in Figure 2D, the normalized intensity in the presence of Hg^2+^ reached a plateau at 90 min, while the normalized intensity in the absence of Hg^2+^ decreased due to the unwanted cleavage reaction at 120 min. Thus, 90 min at which the highest ∆normalized intensity was obtained was selected for the MNAzyme-catalyzed cleavage for further experiments.

### 3.4. Sensitivity and Specificity of the Hg^2+^ Detection Assay

**Figure 3:**
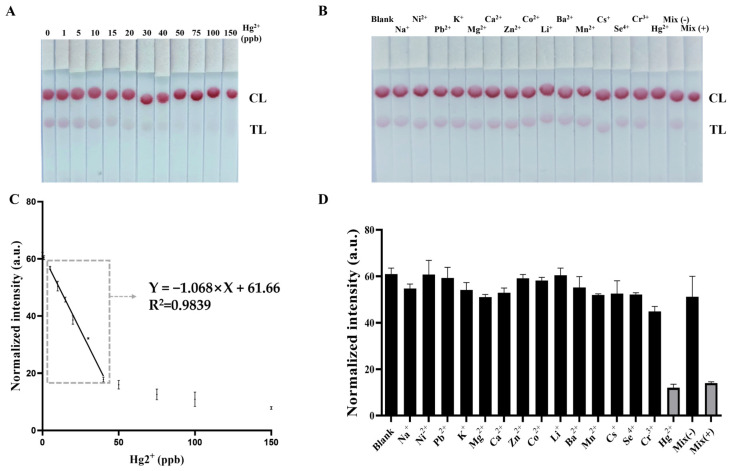
Sensitivity and specificity of the Hg^2+^ detection assay. (**A**) Images of NALFA strips for detection sensitivity. (**B**) Images of NALFA strips for detection specificity. (**C**) Normalized intensity at various concentrations of Hg^2+^ (0, 1, 5, 10, 15, 20, 30, 40, 50, 75, 100, and 150 ppb). (**D**) Normalized intensity in the presence of various metal ions. The concentration of Hg^2+^ was 100 ppb, while the concentration of the other metals was 1000 ppb. Mix (−) and (+) indicate samples containing 14 metal ions (each 100 ppb) without and with Hg^2+^ (100 ppb), respectively. In (**C**,**D**), “Blank” indicates the sample without Hg^2+^. Error bars represent the standard deviation from three independent experiments.

Under the optimized reaction conditions, the colorimetric intensities at different concentrations of Hg^2+^ were measured to evaluate the detection sensitivity for Hg^2+^. In Figure 3A,C, the normalized intensity gradually decreased as the concentration of Hg^2+^ increased, and a linear relationship was observed from 5 ppb to 40 ppb according to the linear regression equation Y = −1.068 × X + 61.66, where X is the concentration of Hg^2+^ (ppb) and 0.9839 is the correlation coefficient. The detection limit was estimated to be 1.874 ppb (detection limit = 3σ per slope) [25,26]. These results confirmed that the developed Hg^2+^ detection assay can detect Hg^2+^ with sufficient sensitivity using a colorimetric signal readout and exhibits comparable or superior performance compared to existing other colorimetric detection methods (Table S2). Furthermore, the effects of another 14 metal ions on the proposed system were investigated. As shown in Figure 3B,D, the presence of other metal ions even at a concentration 10-times higher than that of Hg^2+^ did not influence the proposed system, generating an intense colorimetric signal comparable to that of the blank. In contrast, the presence of Hg^2+^ significantly decreased the normalized intensity, which was observed even in mixed samples where all the other metal ions were present, proving the excellent specificity of the proposed system.

### 3.5. Practical Application of the Hg^2+^ Detection Assay

Finally, the proposed Hg^2+^ detection assay was applied to analyze tap water samples spiked with different concentrations of Hg^2+^. As shown in Table 1, three different concentrations of Hg^2+^ in tap water were determined with good recovery rates ranging from 98.717 to 111.099%, and a low coefficient of variation (CV) of less than 2.688%. These results prove the reliability of the proposed method for the determination of Hg^2+^ in real and environmental samples, demonstrating that the method is highly robust and reproducible.

## 4. Conclusions

We developed a NALFA-based colorimetric assay for the detection of Hg^2+^ using an MNAzyme engineered to contain T-T mismatches. Through in-depth optimization, the proposed assay was able to determine Hg^2+^ down to a concentration of 1.874 ppb with high specificity, which is sufficient to meet the minimum allowable concentration (6 ppb) in water, as set by the WHO standards. Since the detection system relies on a paper-based NALFA system, the presence of Hg^2+^ can be visually confirmed even with the naked eye, and it can be readily deployed in the field, which is highly advantageous over conventional analytical methods, such as ICP-MS, ICP-AES, ICP-OES, CV-AFS, and HPLC. Owing to these characteristics, the proposed Hg^2+^ detection assay is expected to be a useful tool in various fields, such as environmental monitoring and public health. Furthermore, we believe that the system could be applied to not only the detection of other heavy metals but also the simultaneous detection of different heavy metals if the appropriate functional nucleic acids such as DNAzyme or MNAzyme specific to particular heavy metals are uncovered.

## Figures and Tables

**Table 1:** Determination of Hg^2+^ in tap water.

Sample	Added (ppb)	ICP-OES (ppb)	Found (ppb) ^a^	SD ^b^	CV (%) ^c^	Recovery (%) ^d^
Tap water	10	12	11.07	0.30	2.69	92.23
	15	15	16.59	0.35	2.12	110.61
	25	22	24.92	0.42	1.70	113.26

^a^ Mean of three measurements. ^b^ Standard deviation of three measurements. ^c^ Coefficient of variation = SD/mean × 100. ^d^ Found value/ICP-OES × 100.

## Data Availability

Data will be made available on request.

## Supplementary Materials

The following supporting information can be downloaded at: https://www.mdpi.com/article/10.3390/bios14100454/s1, Table S1: Oligonucleotide sequences used in this study; Table S2: Comparison of this method with the previous colorimetric methods for Hg^2+^; Figure S1: Absorbance spectra of AuNPs (solid) and Tag DNA-modified AuNPs (dotted); Figure S2: Schematic illustration of the NALFA strip; Figure S3: Images of the Urea-PAGE gel. Lanes 1–4 are M1 DNA, M2 DNA, the substrate, and the cleaved substrates (I and II; Table S1), respectively, and lanes 5 and 6 are the reaction products where all detection components (M1 DNA, M2 DNA, and substrate) are present without and with Hg^2+^ (100 ppb), respectively; Figure S4: Normalized intensity at different substrate concentrations in the NALFA system. The slope was calculated by dividing the increase in the normalized intensity by each substrate concentration. Error bars represent the standard deviation from three independent experiments. References [27,28,29,30] are cited in the Supplementary Materials.
