# Supplementary material for: MNAzyme-Assisted Nucleic Acid Lateral Flow Assay for Cost-Effective, On-Site Mercury Detection
Source: Biosensors (Basel). 2024 Sep 25;14(10):454. doi: 10.3390/bios14100454 (PMC11505659; doi:10.3390/bios14100454)
Supplement: Supplementary file 1 [file biosensors-14-00454-s001.zip › biosensors-3203102-supplementary.pdf]

Supplementary Information

MNAzyme-Assisted Nucleic Acid Lateral Flow Assay for Cost-Effective, On-Site Mercury Detection

Seok Hyeon Kim, Yujun Kim, Seokjoon Kim, Eun Sung Lee, Byung Seok Cha and Ki Soo Park *

Department of Biological Engineering, College of Engineering, Konkuk University, Seoul 05029, Republic of Korea; sh7041@konkuk.ac.kr (S.H.K.); yujun005@konkuk.ac.kr (Y.K.); ghjghy@konkuk.ac.kr (S.K.); afish94@konkuk.ac.kr (E.S.L.); cbs934@konkuk.ac.kr (B.S.C.)

* Correspondence: akdong486@konkuk.ac.kr or kskonkuk@gmail.com; Tel./Fax: +82-2-450-3742

Table S1. Oligonucleotide sequences used in this study.

Name	Sequence (5′ to 3′)
Substrate	CTC TAG CAT GGA CC**rG rU**CT GAC TGT ATC TC
M1 DNA	CAG TCA GAG GCT AGC TTA TTG TGT TGC TGC
M2 DNA	GCA GCT TCT CTT TAACA ACG AGG TCC ATG
Tag DNA	TTT TTT TCG GTC CAT GCT AGA GAA AAA AA
Capture DNA in the test line	GAG ATA CAG TCA GA
Capture DNA in the control line	CTC TAG CAT GGA CCG
Cleaved substrate (I)	CTC TAG CAT GGA CCG
Cleaved substrate (II)	TCT GAC TGT ATC TC

* Bold letters indicate ribonucleotide and cleavage sites. Red letters indicate the catalytic core of the MNAzyme. Underlined letters indicate the Hg^{2+} recognition sequences.

Table S2. Comparison of this method with the previous colorimetric methods for Hg^{2+}.

**Key detection components**	**Limit of Detection (ppb)**	**Detection Time (min)**	**Reference**
3D-printed rolling circle amplification chip	2.2	60	[1]
G-quadruplex based DNAzyme	10	130	[2]
G-quadruplex based DNAzyme	20	140	[3]
Phosphorothioate based DNAzyme	10	215	[4]
MNAzyme-assisted NALFA	1.874	120	This work

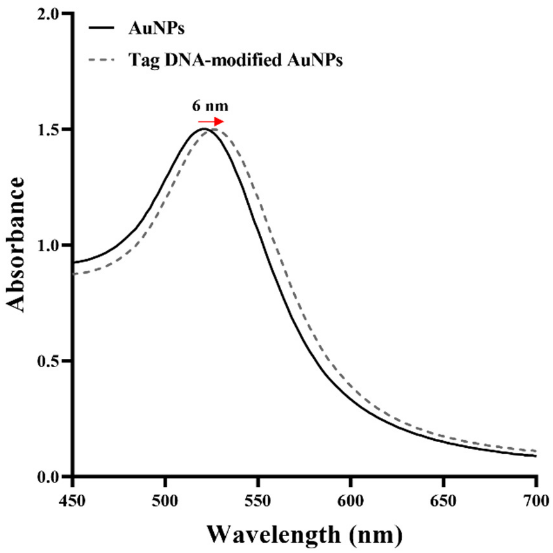

Figure S1. Absorbance spectra of AuNPs (solid) and Tag DNA-modified AuNPs (dotted).

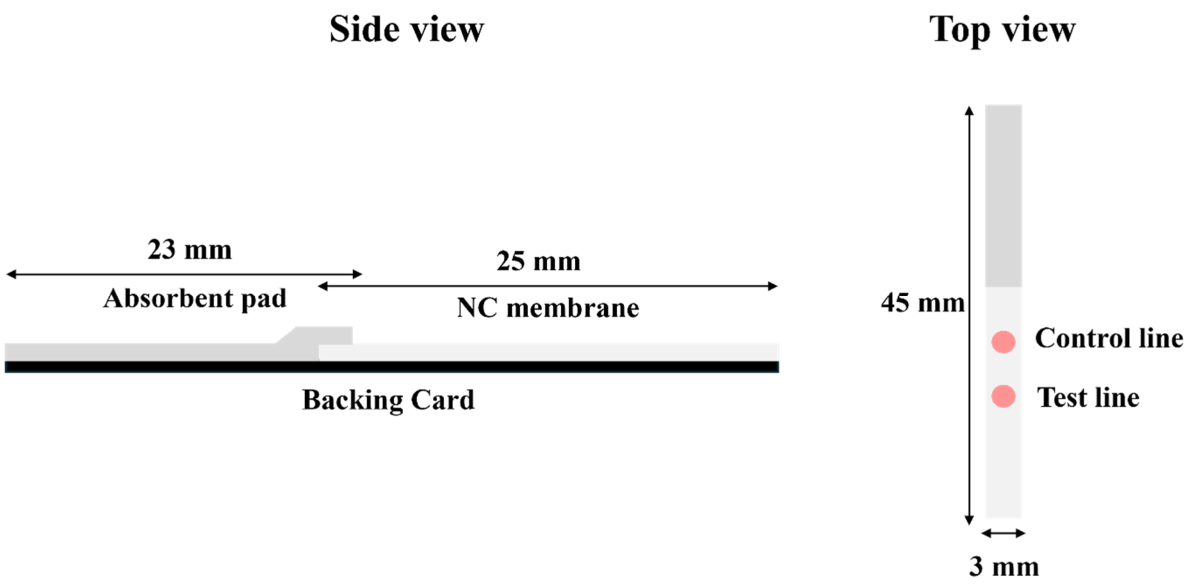

Figure S2. Schematic illustration of the NALFA strip.

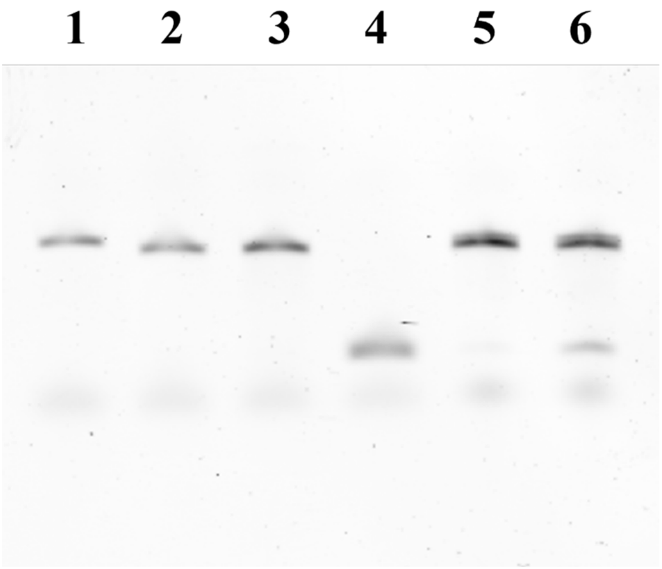

Figure S3. Images of the Urea-PAGE gel. Lanes 1-4 are M1 DNA, M2 DNA, the substrate, and the cleaved substrates (I and II; Table S1), respectively, and lanes 5 and 6 are the reaction products where all detection components (M1 DNA, M2 DNA, and substrate) are present without and with Hg^{2+} (100 ppb), respectively.

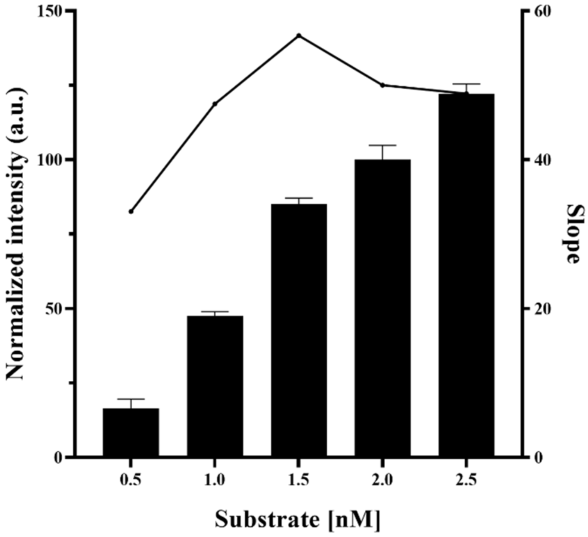

Figure S4. Normalized intensity at different substrate concentrations in the NALFA system. The slope was calculated by dividing the increase of the normalized intensity by each substrate concentration. Error bars represent the standard deviation from three independent experiments.

References

1. Lim, J.W.; Kim, T.Y.; Choi, S.W.; Woo, M.A. 3D-Printed Rolling Circle Amplification Chip for on-Site Colorimetric Detection of Inorganic Mercury in Drinking Water. *Food Chem.* **2019**, 300, 125177.
2. Li, T.; Dong, S.; Wang, E. Label-Free Colorimetric Detection of Aqueous Mercury Ion (Hg2+) Using Hg 2+-Modulated G-Quadruplex-Based Dnazymes. *Anal. Chem.* **2009**, 81, 2144–2149.
3. Li, T.; Li, B.; Wang, E.; Dong, S. G-Quadruplex-Based DNAzyme for Sensitive Mercury Detection with the Naked Eye. *Chem. Commun.* **2009**, 0, 3551–3553.
4. Zhang, D.; Deng, M.; Xu, L.; Zhou, Y.; Yuwen, J.; Zhou, X.; Zhang, D.; Deng, M.; Xu, L.; Zhou, Y.; et al. The Sensitive and Selective Optical Detection of Mercury(II) Ions by Using a Phosphorothioate DNAzyme Strategy. *Chem. - Eur. J.* **2009**, 15, 8117–8120.
